# Race, Ethnicity, and Age Trends in Persons Who Died from COVID-19 — United States, May–August 2020

**DOI:** 10.15585/mmwr.mm6942e1

**Published:** 2020-10-23

**Authors:** Jeremy A.W. Gold, Lauren M. Rossen, Farida B. Ahmad, Paul Sutton, Zeyu Li, Phillip P. Salvatore, Jayme P. Coyle, Jennifer DeCuir, Brittney N. Baack, Tonji M. Durant, Kenneth L. Dominguez, S. Jane Henley, Francis B. Annor, Jennifer Fuld, Deborah L. Dee, Achuyt Bhattarai, Brendan R. Jackson

**Affiliations:** ^1^CDC COVID-19 Emergency Response; ^2^Epidemic Intelligence Service, CDC; ^3^National Center for Health Statistics, CDC; ^4^NYC Health + Hospitals, New York, New York.

During February 12–October 15, 2020, the coronavirus disease 2019 (COVID-19) pandemic resulted in approximately 7,900,000 aggregated reported cases and approximately 216,000 deaths in the United States.* Among COVID-19–associated deaths reported to national case surveillance during February 12–May 18, persons aged ≥65 years and members of racial and ethnic minority groups were disproportionately represented (*1*). This report describes demographic and geographic trends in COVID-19–associated deaths reported to the National Vital Statistics System^†^ (NVSS) during May 1–August 31, 2020, by 50 states and the District of Columbia. During this period, 114,411 COVID-19–associated deaths were reported. Overall, 78.2% of decedents were aged ≥65 years, and 53.3% were male; 51.3% were non-Hispanic White (White), 24.2% were Hispanic or Latino (Hispanic), and 18.7% were non-Hispanic Black (Black). The number of COVID-19–associated deaths decreased from 37,940 in May to 17,718 in June; subsequently, counts increased to 30,401 in July and declined to 28,352 in August. From May to August, the percentage distribution of COVID-19–associated deaths by U.S. Census region increased from 23.4% to 62.7% in the South and from 10.6% to 21.4% in the West. Over the same period, the percentage distribution of decedents who were Hispanic increased from 16.3% to 26.4%. COVID-19 remains a major public health threat regardless of age or race and ethnicity. Deaths continued to occur disproportionately among older persons and certain racial and ethnic minorities, particularly among Hispanic persons. These results can inform public health messaging and mitigation efforts focused on prevention and early detection of infection among disproportionately affected groups.

In NVSS data, confirmed or presumed COVID-19–associated deaths are assigned the *International Classification of Diseases, Tenth Revision* code U07.1 as a contributing or underlying cause of death on the death certificate. The underlying cause of death is the condition that began the chain of events ultimately leading to the person’s death. COVID-19 was the underlying cause for approximately 92% of COVID-19–associated deaths and was a contributing cause for approximately 8% during the investigation period (*2*). NVSS data in this report exclude deaths among residents of territories and foreign countries.

Using NVSS data from May 1 through August 31, 2020, CDC tabulated the numbers and percentages of COVID-19–associated deaths by age, sex, race and ethnicity (categorized as Hispanic, White, Black, non-Hispanic Asian [Asian], non-Hispanic American Indian or Alaska Native [AI/AN], non-Hispanic Native Hawaiian or other Pacific Islander [NHPI], non-Hispanic multiracial [multiracial], and unknown), U.S. Census region,^§^ and location of death (e.g., hospital, nursing home or long-term care facility, or residence). Because only 0.5% of COVID-19 decedents were either NHPI or multiracial, and counts <10 are suppressed in NVSS to maintain confidentiality, these groups were combined into one group for analyses. Age, race and ethnicity, and place of death were unknown for two (<0.01%), 465 (0.4%), and 46 (0.04%) deaths, respectively. To describe changes in demographic features over time, percentages of deaths among two age groups (≥65 years and <65 years), racial and ethnic groups, and U.S. Census region were calculated for each month. R statistical software (version 3.6.3; The R Foundation) was used to tabulate death counts and generate histograms. This activity was reviewed by CDC and was conducted consistent with applicable federal law and CDC policy.^¶^

During May 1–August 31, 2020, a total of 114,411 COVID-19–associated deaths were reported to NVSS (Table). The number of COVID-19–associated deaths decreased from 37,940 in May to 17,718 in June; subsequently, counts increased to 30,401 in July and declined to 28,352 in August. Among decedents, the majority were male (53.3%), White (51.3%), aged ≥65 years (78.2%), and died in an inpatient health care setting (64.3%). Overall, 24.2% of decedents were Hispanic, 18.7% were Black, 3.5% were Asian, 1.3% were AI/AN, and 0.5% were either NHPI or multiracial. During the period studied, the largest percentage of COVID-19–associated deaths occurred in the South Census region (45.7%), followed by the Northeast (20.5%), the West (18.3%), and the Midwest (15.5%). Twenty-two percent of decedents died in a nursing home or long-term care facility.

**TABLE:** Demographic characteristics of persons who died because of COVID-19* (N = 114,411) — National Vital Statistics System (NVSS), United States, May 1–August 31, 2020^†^

Characteristic	Deaths,^§^ %
**Age group, yrs**	
<1	<0.1
1–4	<0.1
5–17	<0.1
18–29	0.5
30–39	1.4
40–49	3.5
50–64	16.4
65–74	21.7
75–84	26.0
≥85	30.4
Unknown	<0.1
**Sex**	
Male	53.3
Female	46.7
Other	0.0
**Race/Ethnicity**	
White, non-Hispanic	51.3
Hispanic or Latino	24.2
Black, non-Hispanic	18.7
Asian, non-Hispanic	3.5
American Indian or Alaska Native, non-Hispanic	1.3
Other, non-Hispanic^¶^	0.5
Unknown race/ethnicity	0.4
**U.S. Census region of residence**	
South	45.7
Northeast	20.5
West	18.3
Midwest	15.5
**Place of death**	
Health care setting, inpatient	64.3
Nursing home or long-term care facility	21.5
Decedent's home	5.2
Hospice facility	3.7
Health care setting, outpatient or emergency department	3.1
Other	2.0
Health care setting, dead on arrival	0.1
Unknown	<0.1

**Abbreviation:** COVID-19 = coronavirus disease 2019.

* Deaths with confirmed or presumed COVID-19, coded to *International Classification of Diseases, Tenth Revision* code U07.1. These data exclude deaths among foreign residents and territories.

^†^ NVSS data from August are incomplete given reporting lags.

^§^ Percentages may not sum to 100 because of rounding. For two (<0.01%) COVID-19 deaths, age was unknown. Sex and region were known for all decedents. For 465 (0.4%) deaths, race or ethnicity were unknown. For 46 (0.04%) deaths, place of death was unknown.

^¶^ Other race/ethnicity includes persons who were non-Hispanic Native Hawaiian or other Pacific Islander or were non-Hispanic multiracial.

During May–August 2020, the percentage of COVID-19–associated deaths occurring in the South increased from 23.4% in May to 62.7% in August, and in the West from 10.6% to 21.4%; the percentages occurring in the Northeast decreased from 44.2% in May to 4.0% in August, and in the Midwest declined from 21.8% to 11.8% (Figure 1). The percentage of decedents aged ≥65 years decreased from 81.8% to 77.6%, and the percentage of deaths occurring in nursing homes or long-term care facilities decreased from 29.8% to 16.6% (Figure 1).

**FIGURE 1:**
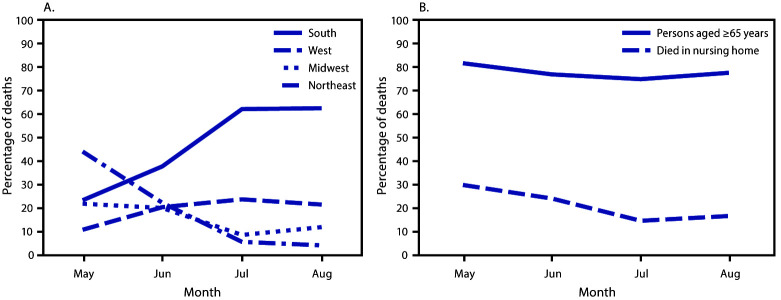
Monthly COVID-19–associated deaths* as a percentage of all deaths, by U.S. Census region, all ages (A), and for persons aged ≥65 years or persons of any age who died in a nursing home or long-term care facility (B) (N = 114,411) — National Vital Statistics System, United States, May 1–August 31, 2020 **Abbreviation:** COVID-19 = coronavirus disease 2019. * Age data were missing for two (<0.01%) COVID-19 deaths, and place of death data were missing for 46 (0.04%) deaths. Total numbers of deaths might vary because of suppression of counts with <10 deaths.

From May to August, the percentage of decedents who were White decreased from 56.9% to 51.5%, and the percentage who were Black decreased from 20.3% to 17.4%, whereas the percentage who were Hispanic increased from 16.3% to 26.4% (Figure 2). Hispanics were the only racial and ethnic group among whom the overall percentage of deaths increased. Among persons aged ≥65 years, the monthly percentage of Hispanic decedents increased in the South (from 10.3% to 21.7%) and West (from 29.6% to 35.4%) and decreased in the Northeast (from 11.3% to 9.3%) and Midwest (from 7.8% to 4.2%). The monthly percentage of Hispanic decedents aged <65 years increased in the South (from 29.2% to 38.1%) and West (from 51.8% to 62.3%) and decreased in the Northeast (from 34.9% to 30.7%) and Midwest (31.1% to 20.4%) (Supplementary Figure, https://stacks.cdc.gov/view/cdc/95229).

**FIGURE 2:**
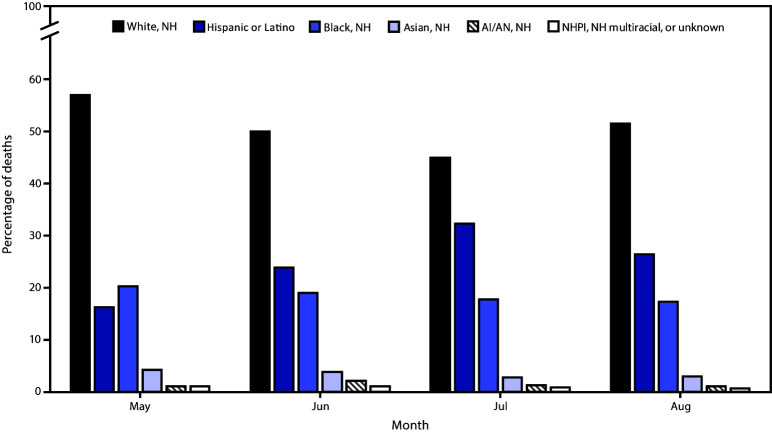
Monthly deaths, by race/ethnicity* as a percentage of all COVID-19–associated deaths (N = 114,411) — National Vital Statistics System, United States, May 1–August 31, 2020 **Abbreviations:** AI/AN = American Indian or Alaska Native; COVID-19 = coronavirus disease 2019; NH = non-Hispanic; NHPI = Native Hawaiian or other Pacific Islander. * Race or ethnicity data were unknown for 465 (0.4%) deaths. Total numbers of deaths might vary because of suppression of counts with <10 deaths.

## Discussion

Based on NVSS data on 114,411 persons who died from COVID-19 in the United States during May–August 2020, the predominant U.S. Census regions shifted from the Northeast to the South and West. The majority of COVID-19–associated deaths occurred among White persons (51.3%), but Black and Hispanic persons were disproportionately represented. Although a small decrease (2.9 percentage points between May and August) in decedents who were Black was observed, Black persons still accounted for 18.7% of overall deaths despite representing just 12.5% of the U.S. population (*3*). Similarly, Hispanic persons were disproportionately represented among decedents: 24.2% of decedents were Hispanic compared with 18.5% of the U.S. population. In addition, the percentage of decedents who were Hispanic increased 10.1 percentage points from May through August. Whereas Hispanic persons accounted for 14% of COVID-19–associated deaths in the United States during February 12–May 18, 2020 (*1*), that percentage increased to approximately 25% in August. Although there has been a geographic shift in COVID-19–associated deaths from the Northeast to the West and South, where Hispanic persons account for a higher percentage of the population, this analysis found that ethnic disparities among decedents in the West and South increased during May–August, 2020, suggesting that the geographic shift alone does not entirely account for the increase in percentage of Hispanic decedents nationwide. Disparities in COVID-19 incidence and deaths among Hispanic persons and other underrepresented racial and ethnic groups are well documented (*4*–*6*) and might be related to increased risk for exposure to SARS-CoV-2, the virus that causes COVID-19. Inequities in the social determinants of health can lead to increased risk for SARS-CoV-2 exposure among some racial and ethnic groups. For example, persons from underrepresented racial and ethnic groups might be more likely to live in multigenerational and multifamily households, reside in congregate living environments, hold jobs requiring in-person work (e.g., meatpacking, agriculture, service, and health care), have limited access to health care, or experience discrimination (*5*,*6*). Differences in the prevalence of underlying conditions (e.g., diabetes and obesity) among racial and ethnic groups might also be associated with increased susceptibility to COVID-19–associated complications and death (*4*).

The shift in COVID-19–associated deaths during May–August 2020 from the Northeast (where 17.1% of the U.S. population resides) into the South and West (where 38.3% and 23.9% of the U.S. population resides, respectively)** is consistent with recent findings documenting the emergence of COVID-19 hotspots^††^ in these regions during June–July 2020 (*7*). The decreasing percentage of deaths occurring among persons aged ≥65 years and persons in nursing homes, which were important sites of COVID-19–associated deaths early in the pandemic, suggests a continued shift toward noninstitutionalized and younger populations. The observed geographic shifts in COVID-19–associated deaths might be related to differential implementation of community mitigation efforts throughout the nation, including earlier reopening efforts in selected jurisdictions. To prevent the spread of COVID-19, CDC continues to recommend the use of masks, frequent handwashing, and maintenance of social distancing, including avoidance of large gatherings (*8*).

The findings in this report are subject to at least two limitations. First, NVSS provisional death data are continually updated and subject to delays. Therefore, this report likely underestimates the number of deaths that occurred, particularly during August 2020, for which data are less complete than previous months. Furthermore, in focusing only on COVID-19–associated deaths captured by NVSS, this report did not address long-term morbidity faced by some persons who survive COVID-19 infections, nor does it account for deaths and morbidity related to the indirect effects of interrupted health care and socioeconomic disruption caused by the pandemic (*9*). For example, one report indicated that by June 30, 2020, an estimated 41% of U.S. adults had delayed or avoided medical care because of concerns about the pandemic, including 12% who reported having avoided urgent or emergency care (*10*).

Despite these limitations, this report provides information on how demographic and geographic factors have changed among COVID-19–associated deaths during May–August 2020. Racial and ethnic disparities among COVID-19 decedents have persisted over the course of the pandemic and continue to increase among Hispanic persons. These results can inform public health messaging and mitigation efforts focused on prevention and early detection of infection among disproportionately affected groups so as to minimize subsequent mortality.

**Summary**

**What is already known about this topic?**

Persons aged ≥65 years and members of minority racial and ethnic groups are disproportionately represented among COVID-19–associated deaths.

**What is added by this report?**

Analysis of 114,411 COVID-19–associated deaths reported to National Vital Statistics System during May–August 2020, found that 51.3% of decedents were non-Hispanic White, 24.2% were Hispanic or Latino (Hispanic), and 18.7% were non-Hispanic Black. The percentage of Hispanic decedents increased from 16.3% in May to 26.4% in August.

**What are the implications for public health practice?**

These results can inform public health messaging and mitigation efforts focused on prevention and early detection of infection among disproportionately affected groups so as to minimize subsequent mortality.

## Footnotes

* CDC official counts of cases and deaths, released daily at https://covid.cdc.gov/covid-data-tracker/, are aggregate counts from reporting jurisdictions. Throughout the COVID-19 pandemic, and separately from the NVSS, CDC has been tracking both aggregate and individual (i.e., line-listed) counts of cases and deaths. For aggregate counts, from January 22 to March 2, 2020, CDC provided laboratory confirmation for all U.S. confirmed cases. Starting March 3, jurisdiction partners validated aggregate counts each night for report released at 12 p.m. the following day by CDC. For individual counts, jurisdiction partners electronically submit standardized information for individual cases of COVID-19 to CDC. From April 14, aggregate and individual counts included confirmed and probable cases and deaths, according to the Council of State and Territorial Epidemiologists (CSTE) position statement Interim 20-ID-01 (https://cdn.ymaws.com/www.cste.org/resource/resmgr/2020ps/interim-20-id-01_covid-19.pdf; https://wwwn.cdc.gov/nndss/conditions/coronavirus-disease-2019-covid-19/case-definition/2020/). On August 5, CSTE published an updated position statement, Interim 20-ID-02, to clarify the interpretation of antigen detection tests and serologic test results within the case classification (https://wwwn.cdc.gov/nndss/conditions/coronavirus-disease-2019-covid-19/case-definition/2020/08/05/).

† https://www.cdc.gov/nchs/nvss/deaths.htm.

§ U.S. Census Bureau regions are Northeast, Midwest, South, and West. https://www2.census.gov/geo/pdfs/maps-data/maps/reference/us_regdiv.pdf.

¶ See e.g., 45 C.F.R. part 46.102(l)(2), 21 C.F.R. part 56; 42 U.S.C. §241(d); 5 U.S.C. §552a; 44 U.S.C. §3501 et seq.

**
https://www.census.gov/popclock/print.php?component=growth&image=//www.census.gov/popclock/share/images/growth_1561939200.png .

†† Counties defined as hotspot counties met all four of the following criteria, relative to the date assessed: 1) >100 new COVID-19 cases in the most recent 7 days, 2) an increase in the most recent 7-day COVID-19 incidence over the preceding 7-day incidence, 3) a decrease of <60% or an increase in the most recent 3-day COVID-19 incidence over the preceding 3-day incidence, and 4) the ratio of 7-day incidence/30-day incidence exceeds 0.31. In addition, hotspots must have met at least one of the following criteria: 1) >60% change in the most recent 3-day COVID-19 incidence or 2) >60% change in the most recent 7-day incidence.
